# Steroid-Resistant Focal Segmental Glomerulosclerosis with Alport-like Glomerular Basement Membrane Lesions Due to a *MYO1E* Mutation: A Pediatric Case Report

**DOI:** 10.3390/ijms27062838

**Published:** 2026-03-20

**Authors:** Andrea Angioi, Doloretta Piras, Nicola Lepori, Paola Bianco, Matteo Floris, Gianfranca Cabiddu, Antonella Barreca, Antonello Pani

**Affiliations:** 1 Nephrology, Dialysis and Transplantation Unit, “Giuseppe Brotzu” Hospital, 09121 Cagliari, Italy; 2 Department of Medical Science and Public Health, University of Cagliari, 09124 Cagliari, Italy; nicola.lepori@aob.it (N.L.); matteo.floris@aob.it (M.F.); gianfrancacabiddu@aob.it (G.C.); antonellopani@aob.it (A.P.); 3 Division of Pathology, “Giuseppe Brotzu” Hospital, 09121 Cagliari, Italy; paola.bianco@aob.it; 4 Pathology Unit, Città della Salute e della Scienza di Torino University Hospital, 10126 Turin, Italy; antonella.barreca@libero.it

**Keywords:** focal segmental glomerulosclerosis, steroid-resistant nephrotic syndrome, *MYO1E*, podocyte cytoskeleton, autosomal recessive inheritance, pediatric nephrology, electron microscopy, genetics

## Abstract

Steroid-resistant nephrotic syndrome (SRNS) in childhood frequently reflects monogenic podocytopathies in which immunosuppression is ineffective. Biallelic variants in *MYO1E*, encoding the class I myosin Myo1E, cause a distinctive form of focal segmental glomerulosclerosis (FSGS) often accompanied by “Alport-like” multilamination of the glomerular basement membrane (GBM). Early recognition has therapeutic and prognostic implications. A previously healthy 4-year-old boy presented with generalized edema and nephrotic-range proteinuria. Glucocorticoids induced no remission; sequential calcineurin inhibition (cyclosporine, then tacrolimus) and a single dose of ofatumumab yielded only transient, partial reductions in proteinuria. A first biopsy elsewhere showed FSGS with nonspecific IgM/C3 trapping; electron microscopy (EM) was not performed. At age 10, repeat biopsy with EM revealed ~30% segmental foot-process effacement, focal GBM thickening (to 1740 nm), irregular lamina densa multilamination, and lamellar duplications without immune-complex deposits—features highly suggestive of hereditary GBM disease. Targeted sequencing identified compound-heterozygous *MYO1E* variants segregating in trans: a canonical splice-donor change (c.2785+1G>A) and a frameshift (c.3094_3097del; p.Thr1032Profs*73). Each parent was an unaffected heterozygous carrier; the sibling was negative. Supportive therapy with ramipril was continued. At last follow-up (January 2025), renal function was normal (serum creatinine 0.5 mg/dL; creatinine clearance 122 mL/min) with stable sub-nephrotic proteinuria (0.52 g/day; 16 mg/m^2^ per hour) and normotension. This case broadens clinicopathologic recognition of *MYO1E*-associated nephropathy and highlights the teaching point that Alport-like GBM changes are not pathognomonic for type IV collagen disorders but may signal defects in podocyte cytoskeletal anchoring.

## 1. Introduction

Focal and Segmental Glomerulosclerosis (FSGS) is a general term that describes a morphological pattern of injury characterized by the occlusion of a single glomerular capillary loop or a group of loops by sclerotic material, reflecting the final stage of a healing process that directly or indirectly involves the podocyte. A diverse range of injury pathways, including autoimmune, genetic, adaptive, infectious, and local inflammation, converge to create an FSGS lesion [1]. Genetic forms of FSGS are challenging to treat because they are usually resistant to glucocorticoids and often progress to end-stage kidney disease (ESKD). Immunosuppressive therapies, including steroids, are generally ineffective in genetic FSGS, making early identification and alternative management crucial [2,3].

Myosin 1E (Myo1E), a nonmuscle class I myosin, is localized to the slit diaphragm. In podocytes, the precise junctional targeting of Myo1E relies on coordinated, multi-domain interactions. The tail homology-1 (TH1) domain binds phosphoinositide lipids in the plasma membrane, anchoring the molecule at the cell cortex. Conversely, the proline-rich TH2 domain interacts with junction-associated scaffold proteins to stabilize its position. Simultaneously, the motor head domain transduces force by interacting with F-actin filaments, and the Src-homology-3 (SH3) domain binds proline-rich partners such as ZO-1, thus promoting the coordinated assembly of cell–cell junctions and associated actin bundles [4].

In murine models, genetic ablation of Myo1E leads to proteinuria, glomerulosclerosis, and basement membrane abnormalities that mirror human disease [5]. Biallelic mutations in *MYO1E* have been identified in familial, childhood-onset FSGS, which is characterized by “Alport-like” multilamination of the glomerular basement membrane and resistance to corticosteroids and calcineurin inhibitors [6].

We describe a novel *MYO1E* mutation in a 10-year-old patient with refractory FSGS, correlating genetic findings with electron microscopic evidence to inform diagnosis and guide treatment.

## 2. Case Report

We report a 10-year-old Caucasian boy who presented at another institution at the age of 4 with generalized edema and nephrotic syndrome. His family history was negative for kidney disease, and his parents were healthy (his father had a history of kidney stones). Since November 2016, initial therapy included high-dose prednisone (60 mg/m^2^), which he continued for two months, then was combined with cyclosporine A (January 2017), resulting in partial remission but persistent proteinuria (0.7 g/24 h). At that time, hypertension was diagnosed, and a low dose of ACE inhibitor was added, showing benefit. As blood tests indicated dyslipidemia, atorvastatin was initiated. After two months of adding cyclosporine and ramipril, an increase in proteinuria was observed (1.9 g/24 h).

In May 2017, a kidney biopsy revealed 3/10 hyaline glomeruli, with 3/6 glomeruli exhibiting segmental sclerosis, perihilar capsular adhesion, and mesangial hypercellularity on light microscopy. Immunofluorescence indicated nonspecific IgM (++−) and C3 (+−−) deposition in capillary walls and mesangial areas. At that time, electron microscopy was not requested. Consequently, cyclosporine was discontinued, and a second-line treatment with tacrolimus was initiated; however, after 6 months, there was no clinical improvement, and proteinuria remained stable at 2.1 g/24 h.

In October 2017, a third line of B-cell-depleting therapy with a single dose of ofatumumab was administered, but it did not significantly improve the clinical pattern (from October 2018 to March 2019: sCr 0.4 mg/dL; 24 h proteinuria between 0.72 and 1.92 g/24 h; serum albumin 3.6 g/dL). Growth and kidney function remained stable (weight 25–50°; height 50–75°), but reliance on immunosuppression raised concerns about cumulative toxicity; thus, no other immunosuppressive treatments were considered, and he continued on a low dose of ramipril.

In early 2021, at the age of 8, he was evaluated in our division. At that time, he had achieved partial remission of his nephrotic syndrome, normal kidney function (eGFR 110 mL/min), proteinuria consistently below 40 mg/m^2^ per hour, and a serum albumin level of 3.5 g/dL. Despite this apparent improvement, subsequent follow-ups revealed a proteinuria of 500–800 mg per 24 h (20–35 mg/m^2^ per hour), while serum albumin remained at 4.0 g/dL. Growth parameters were appropriate for his age, and he showed no clinical signs or symptoms of volume overload.

**Figure 1:**
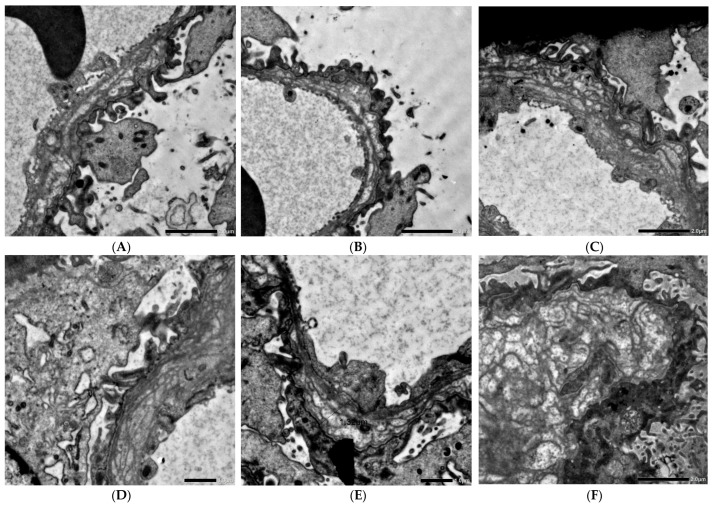
Alport-like ultrastructural changes of glomerular basement membranes. The glomerular capillaries show marked irregular thickening of the GBM (**A**,**B**) and different diameters (up to 1740 nm, (**C**)), mostly preserved foot processes (**A**,**D**), and scalloping on the subepithelial side of the GBM, with multiple electron-lucent areas and multilaminations (**E**,**F**). ((**A**–**C**,**F**) original magnification ×4000, (**D**,**E**) original magnification ×5000, scale bars are shown).

On 22 June 2022, he underwent a second kidney biopsy primarily to obtain tissue for ultrastructural assessment and to quantify the progression of glomerular injury since his initial biopsy. Electron microscopy was performed on one representative glomerulus (Figure 1). Podocyte foot-process effacement was estimated using a semiquantitative approach based on the proportion of capillary loop length demonstrating fusion of foot processes across multiple ultrastructural fields, resulting in an estimated segmental effacement of approximately 30%. The glomerular basement membrane exhibited focal thickening (up to 1740 nm), irregular multilamination of the lamina densa, and areas of pronounced lamellar duplication with occasional intramembranous electron-dense microparticles (Figure 1B). There was also scalloping on the subepithelial side of the GBM with focal abnormal podocyte insertion. In less affected regions, the basement membrane thickness was within normal limits for age or mildly thinned (mean ~195 nm). Capillary lumina were patent, and mesangial architecture was preserved without mesangial electron-dense deposits. No immune complex–type deposits were identified. These ultrastructural features (segmental foot-process fusion and “Alport-like” basement-membrane alterations) strongly suggested a hereditary GBM disease and prompted targeted genetic analysis (Figure 1). The ultrastructural appearance raised suspicion of a *MYO1E* mutation, as previously described in the literature [6]. In light of the limited extent of podocyte foot-process effacement, he was managed conservatively without additional immunosuppressive therapy.

In the genetic analysis, the proband carried two rare predicted disruptive *MYO1E* alleles in trans: a canonical splice-donor mutation (c.2785+1G>A) and a four–base-pair deletion (c.3094_3097del; p.Thr1032Profs*73). Sanger sequencing using a 3730 DNA Analyzer (Applied Biosystems, Waltham, MA, USA) was performed on genomic DNA to analyze the regions harboring these variants in the *MYO1E* gene (NM_004998.4).

Segregation analysis demonstrated that the patient’s father was heterozygous for the c.3094_3097del variant and negative for c.2785+1G>A, whereas the mother carried the c.2785+1G>A variant and was negative for c.3094_3097del, confirming that the two variants are located on opposite alleles (in trans) and supporting a compound heterozygous state consistent with autosomal recessive inheritance. The patient’s sibling did not carry either variant.

The frameshift variant c.3094_3097del (p.Thr1032Profs*73) has been previously reported in the Human Gene Mutation Database (HGMD; accession CD156544) and is classified as likely pathogenic in ClinVar (RCV001328212). Population frequency data obtained from the Genome Aggregation Database (gnomAD) indicate that both variants are either absent or extremely rare in the general population, supporting their potential disease association.

The splice-site variant c.2785+1G>A affects the invariant +1 position of the donor splice site of intron 24 and is predicted to disrupt normal mRNA splicing. In silico prediction tools, including Franklin (Genoox, Palo Alto, CA, USA) and Alamut Visual Plus (SOPHiA GENETICS, Bidart, France), consistently classify this variant as pathogenic or likely pathogenic due to its predicted effect on canonical splice-site function.

**Figure 2:**
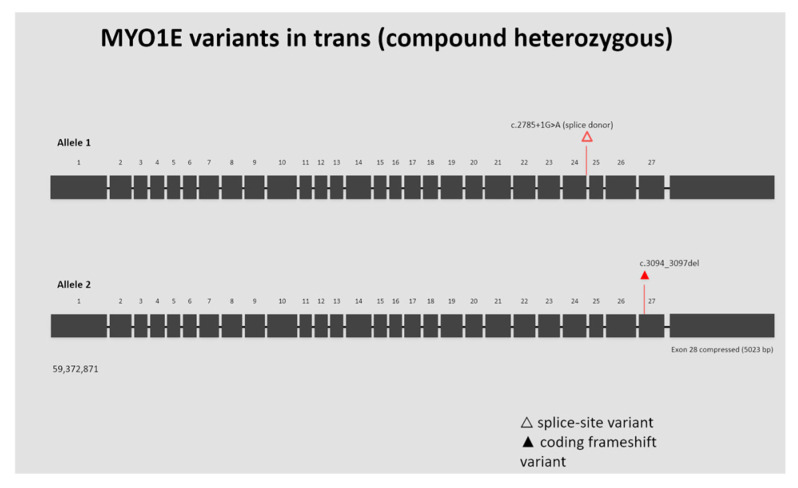
Exons are shown as boxes and introns as lines (not to scale). Exon 28 is compressed for visualization. The splice-site variant c.2785+1G>A (open triangle) occurs at the exon 24–intron 24 boundary. The frameshift variant c.3094_3097del (triangle) lies within exon 27. The variants are present in trans on the two *MYO1E* alleles.

Both variants affect highly conserved regions of the *MYO1E* gene and are predicted to lead to loss of function of the Myo1E protein, either through nonsense-mediated mRNA decay (splice-site mutation) or through truncation of the C-terminal tail domain caused by the frameshift variant (Figure 2).

Importantly, both parents, each heterozygous for one variant, were clinically asymptomatic and free of proteinuria, supporting a recessive loss-of-function disease mechanism consistent with previously described *MYO1E*-associated nephropathy. At the most recent follow-up (January 2025), the patient showed normal renal function (serum creatinine 0.5 mg/dL; creatinine clearance 122 mL/min), proteinuria of 524 mg/24 h (16.2 mg/m^2^/h), and serum albumin of 3.7 g/dL. He was on treatment with ramipril 3.75 mg/day and had blood pressure values within the optimal range for age.

## 3. Discussion

Although rare, highly penetrant variants in established podocyte genes account for roughly one-third of FSGS cases [7]. *MYO1E* mutations are part of this contest. They present as a rare, autosomal recessive cause of FSGS, typically surging in childhood and often associated with steroid-resistant nephrotic syndrome.

Myo1E is part of the class 1 myosins, which are associated with processes that require membrane deformation, such as endo- and exocytosis and phagocytosis [8]. They are characterized by their ability to bind membrane phospholipids through the positively charged region in their tail domain, known as the tail homology 1 (TH1). Therefore, Myo1E is typically associated with the plasma membrane or membrane-bound organelles such as endosomes, the Golgi apparatus, and endocytic or exocytic vesicles [8,9].

In podocytes, Myo1E is a crucial component of the slit diaphragm complex. It is designed for quick sliding in areas with high cross-linked actin filaments, exhibiting low actin affinity and rapid phosphate release [10]. The study of *MYO1E* gene mutations helped the domain-mapping process. The motor domain plays a crucial role in actin cable formation; TH1 and TH2 are essential targeting modules that bind to membrane phospholipids, while SH3 domains mediate effector interactions, notably with ZO-1 and synaptopodin [5,9].

In our patient, the c.2785+1G→A substitution at the invariant +1 splice-donor site of intron 24 is predicted to abolish normal excision of the exon 24–25 junction, yielding aberrant transcripts that undergo nonsense-mediated decay and effectively eliminate Myo1e protein production from that allele [11]. Concurrently, the four-base-pair deletion c.3094_3097del in exon 27 shifts the reading frame at Thr1032 and introduces a premature stop codon, truncating the C-terminal tail domain and thereby removing the lipid-binding TH1 motif and adjacent SH3-like region necessary for membrane anchorage and actin-cytoskeleton tethering [8]. The combined loss of Myo1e’s motor-head autoinhibition interface (via haploinsufficiency) and its tail-mediated linkage to the podocyte plasma membrane disrupts dynamic actin remodeling at foot processes, precipitating foot-process effacement, slit-diaphragm breakdown, and the focal segmental glomerulosclerosis phenotype observed [11,12]. These abnormalities in podocytes and the glomerular basement membrane, including distinctive diffuse and global Alport-like GBM changes and podocyte damage [6,12,13], contribute to chronic inflammation and scarring [12].

Most of the pathogenic *MYO1E* variants are located in the protein’s motor and neck domains. They are typically reported as homozygous mutations in consanguineous families, although compound heterozygotes have also been identified. FSGS-associated mutations in Myo1 in fission yeast disrupt myosin localization and function, resulting in differential effects on protein stability [14]. Moreover, *MYO1E* may play a role in immunity, as it is critical for the adhesion and migration of activated B cells at high endothelial venules, regulating integrins and the PI3K-FAK-RAC-1 signaling pathway [15], but also influencing an efficient neutrophil extravasation and adhesive interactions with the vascular endothelium [16].

Patients with *MYO1E* mutations usually present with steroid-resistant nephrotic syndrome between the ages of 1 and 11, and some progress to kidney failure within 4 to 10 years. Most cases of genetic FSGS, including those caused by *MYO1E* mutations, do not respond to standard immunosuppressive therapy. Some reports support calcineurin inhibitors (cyclosporine A) and low-dose glucocorticoid treatment, which effectively reduce proteinuria and permit a partial remission [13], but the literature is contradictory [6]. Moreover, our patient’s proteinuria demonstrated marked fluctuations, ranging from approximately 2.5 g/day to 0.6 g/day, mirroring patterns previously described in patients treated with calcineurin inhibitors. This oscillation likely reflects a dual mechanism: transient afferent arteriolar vasoconstriction, which reduces glomerular perfusion and GFR, coupled with podocyte cytoskeletal remodeling secondary to calcineurin inhibition, leading to reversible modulation of filtration-barrier permeability. On the other hand, supportive therapy with ACEi is widely adopted in these patients to counteract adaptive changes in the glomeruli [6].

Immunofluorescence staining for collagen IV α-chains was not available at our institution at the time of the biopsy. Consequently, the diagnostic workup relied on ultrastructural examination and subsequent genetic testing.

## 4. Conclusions

This case report of a pediatric patient with steroid-resistant FSGS due to a confirmed *MYO1E* mutation, supported by characteristic Alport-like glomerular basement membrane lesions on electron microscopy, significantly contributes to understanding *MYO1E* nephropathy. The distinct ultrastructural findings provide a valuable diagnostic indicator for clinicians and pathologists, suggesting a broader role for Myo1E in maintaining GBM integrity beyond its established function in podocyte cytoskeleton dynamics. The case underscores the imperative for early and comprehensive diagnostic evaluation, including genetic testing and electron microscopy, in all children presenting with steroid-resistant FSGS. Such an approach facilitates accurate diagnosis, guides appropriate management strategies, and improves patient outcomes by avoiding unnecessary treatments and providing precise prognostic information.

## Data Availability

The data presented in this study are not publicly available due to privacy and ethical restrictions but are available from the corresponding author upon reasonable request.
